# Organotypic brain slice cultures to model neurodegenerative proteinopathies

**DOI:** 10.1186/s13024-019-0346-0

**Published:** 2019-12-02

**Authors:** C. L. Croft, H. S. Futch, B. D. Moore, T. E. Golde

**Affiliations:** 1 Department of Neuroscience, College of Medicine, University of Florida, Gainesville, FL 32610 USA; 2 Center for Translational Research in Neurodegenerative Disease, College of Medicine, University of Florida, Gainesville, FL 32610 USA; 3 McKnight Brain Institute, College of Medicine, University of Florida, Gainesville, FL 32610 USA

**Keywords:** Amyloid-β, Organotypic brain slice cultures, Tau, Neurodegeneration, Proteinopathies, Alzheimer’s disease, Microglia, Recombinant adeno-associated virus

## Abstract

Organotypic slice cultures of brain or spinal cord have been a longstanding tool in neuroscience research but their utility for understanding Alzheimer’s disease (AD) and other neurodegenerative proteinopathies has only recently begun to be evaluated. Organotypic brain slice cultures (BSCs) represent a physiologically relevant three-dimensional model of the brain. BSCs support all the central nervous system (CNS) cell types and can be produced from brain areas involved in neurodegenerative disease. BSCs can be used to better understand the induction and significance of proteinopathies underlying the development and progression of AD and other neurodegenerative disorders, and in the future may serve as bridging technologies between cell culture and in vivo experiments for the development and evaluation of novel therapeutic targets and strategies. We review the initial development and general use of BSCs in neuroscience research and highlight the advantages of these cultures as an ex vivo model. Subsequently we focus on i) BSC-based modeling of AD and other neurodegenerative proteinopathies ii) use of BSCs to understand mechanisms underlying these diseases and iii) how BSCs can serve as tools to screen for suitable therapeutics prior to in vivo investigations. Finally, we will examine i) open questions regarding the use of such cultures and ii) how emerging technologies such as recombinant adeno-associated viruses (rAAV) may be combined with these models to advance translational research relevant to neurodegenerative disorders.

## Background

The cellular and molecular complexity of the brain and spinal cord has presented challenges for ex vivo studies of the central nervous system (CNS). Though much has been learned, and will continue to be learned from future studies, of isolated primary brain cells, these “reductionist” methodologies have major limitations and can be highly misleading [1]. An excellent illustration of confounds introduced by isolated cultures of specific cell types are studies showing that cultured primary mouse microglia are highly phenotypically distinct from the resident microglia in the brain [2]. Though human induced pluripotent stem cell (iPSC)-derived mini-brain organoids hold promise for studying these complex interactions, these studies still face many technical and conceptual challenges [3]. Further, because of costs and complexity, iPSC-derived brain organoids are not widely accessible to the broader research community.

Though rodent acute brain slices have been used for many years, primarily for electrophysiology studies [4], long-term organotypic brain slice cultures (BSCs) have emerged as a relatively straightforward, inexpensive and easily implemented, method to study a cytoarchitecturally intact 3D model of the brain ex vivo [1]. Here we discuss i) the origins of BSC methodology, ii) the advantages and limitations of these models, and iii) how they have been used to study CNS proteinopathies. Finally we describe i) recent advances from our laboratory with respect to using recombinant adeno-associated viral (rAAV) vectors to transduce and model select neurodegenerative pathologies in these cultures and ii) discuss how these BSCs might be used in the future to accelerate both our understanding of CNS diseases and as technology accelerators to identify and validate novel therapeutic strategies.

## Organotypic brain slice cultures

The use of BSCs of postnatal rodent brain to model physiological and developmental properties was first widely established with the introduction of the roller-tube method in the 1980s [5]. However, tissue cultured in this manner does not preserve the cytoarchitecture of the region of interest and was superseded by the development of the interface-slice culture method in the 1990s [6]. The interface method involves culturing the tissue region of interest on a porous membrane interface between a humidified atmosphere and the culture medium. The explants of tissue attach to the membrane and receive nutrition from the slice culture medium through the membrane via capillary action. Since the introduction of the membrane-interface slice culture method, the use of BSCs in neuroscience research has increased dramatically [7] and the majority of studies discussed in this review prepare BSCs in this manner.

As for acute BSCs frequently used for electrophysiological studies; the most commonly cultured brain region for long-term cultures in neurodegeneration research is the hippocampus, as this region is typically affected by neuronal loss and related pathologies in multiple neurodegenerative conditions. Various other regions of the brain including cortex, cerebellum, and thalamus have been successfully cultured [8–10]. In addition co-cultures of various brain regions including the nigrostriatal circuit [11], the cortex and hippocampus [12, 13], as well as, entire sagittal and coronal BSCs [14, 15] have been reported.

Cell death and viability in BSCs is assessed by several methods including staining of living or dead cells using propidium iodide or other similar stains, by measuring the release of lactate dehydrogenase into the culture medium or by measuring metabolic activity [12, 16, 17]. The majority of BSCs are typically produced from mice or rats up to postnatal day 12; at this age the cytoarchitecture is established, the brain is larger and easier to manipulate and neuronal cells are likely to survive explantation [9]. These BSCs also display high levels of plasticity and therefore show resistance to the mechanical trauma incurred when neuronal processes are cut [18]. There are far fewer reports of BSCs kept for more than a few hours from adult animals as most neuronal cells within these slices do not survive for any appreciable length of time. Nevertheless, following significant alterations to the traditional protocol including culturing at lower temperatures, different culture mediums, carbogen atmospheres and reducing the thickness of slices some adult BSCs have been reported, however extensive cell loss is still apparent [19–21]. In addition, cultures prepared from human brain biopsies, post-mortem brains or embryonic brains have been reported [22–25]. These have their own limitations including, but not limited to, ethical considerations, availability of tissue and post-mortem delay affecting the success of culture [24]. Recent work combining rAAVs with human BSCs over long-term culture will also likely prove promising for future neurodegeneration studies [26].

## Advantages of BSCs as a model system

A main advantage of BSCs is that they retain a three-dimensional organization in culture and are, at least partially, anatomically intact and representative of the area of which they are derived [9]. Despite spherical ‘organoids’ emerging as promising models for neurodegeneration research [3], they do not have the anatomical planes of connectivity lending BSCs to be a more appropriate approach when a maintained anatomy is required. Indeed, network connectivity studies have been conducted in hippocampal BSCs [27] and differences between hippocampal and cortical networks have been identified in these BSCs [28]. Neuronal and non-neuronal cells from the brain grown ex vivo in BSCs are representative of the populations found in vivo [8, 14, 29]. This is highly important in the study of most neurodegenerative diseases where changes in cell types other than neurons are implicated; BSCs contain glia in similar proportions as observed in vivo [21, 30, 31]. Additionally, these BSCs maintain vascular cells [1, 32]. This system therefore allows all cell types in the brain to be studied in an anatomically preserved environment. This is not the case when using dissociated cultures, primary cell lines or iPSC cultures.

A further advantage of this ex vivo system is that the development of cells and synapses in ex vivo BSCs mimics the development of the brain in vivo [18]. Neurons morphologically develop ex vivo as they do in vivo in acute preparations and retain similar synaptic connectivity, intact neuronal function and circuitry as observed in intact brain. Indeed, organotypic hippocampal BSCs maintain electrical properties comparable to those observed in acute slices [33] and synapses mature in culture towards a phenotype recapitulative of adult brain [9, 18]. Developmental changes in spine density and shape and increased connectivity recapitulate the in vivo phenotype observed in acute slices from age-matched time points [18, 34]. Similarly, select genes and proteins expressed in BSCs have been shown to be comparable to in vivo expression levels [9, 35]. With the development of proteomic and transcriptomic analysis methods, it would also be interesting to obtain a comprehensive analysis of global gene and protein levels in BSCs compared to in vivo tissues.

BSCs can be used as an alternative to some in vivo experiments thereby reducing the number of animals required for certain studies and precluding the need to age multiple animals as is required for many neurodegeneration studies [36]. As multiple BSCs are prepared from one animal, this allows the investigation of several variables in one system, potentially reducing variability. At least 36 individual sections of 350 μm thickness can be generated from a single P8/9 mouse, allowing multiple compounds or observations of several disease-related changes over time to be investigated at the same time in tissue from the same animal [29, 37] . In addition, a further benefit to the use of postnatal BSCs prior to weaning is that smaller colonies of mice can be kept thus saving time, money and the requirement of aged mice with severe phenotypes [36]. Indeed, as will be discussed later, pathology in BSCs can be highly accelerated*.*

In addition, BSCs can be readily manipulated by the addition of compounds, viral tools, anti-sense oligonucleotides and other approaches into the culture medium or small volumes directly on to the slices [29, 37]. This is further permitted by the absence of a blood-brain-barrier providing direct access to confirm target engagement in the brain. Lastly, techniques that may be typically difficult to execute in vivo such as long-term live imaging or electrophysiology can be applied to BSCs.

## Limitations of BSCs to study neurodegenerative proteinopathies

Despite the aforementioned advantages of BSC models, there are some limitations to their study in neurodegenerative proteinopathies. At the present time there are only a few examples of observations made in BSC models, which i) were subsequently validated in vivo or ii) validated an observation previously made in vivo [1, 12, 13, 37, 38]. Thus, until there are more examples of the predictive value of BSC models with respect to subsequent in vivo studies, BSC models are best viewed as bridging experiments. Indeed, BSCs can potentially provide cost-effective models to help screen a modest list of therapeutic candidates or genetic manipulations [29], but unless they become more automated it is unlikely that one can envision using them to screen more than 50–100 experimental manipulations at a time. The BSC models discussed in the following sections are prepared from rodent brain, which of course have the same general limitations as do in vivo rodent models [39]. Most BSC models rely on the overexpression of wild-type or mutant human genes, and thus have similar limitations as the transgenic overexpression models [39]. Genetic overexpression generally provides a rapid development of pathology which facilitates the study of many of these neurodegenerative proteinopathies. At least for studies of amyloid-β (Aβ) and α-synuclein pathology, overexpression of wild type alleles can result in disease in humans, thus, these studies do have some face validity. Pathology is not typically induced in the absence of overexpression, except when exogenous proteinopathic seeds are applied and used to drive pathology. Both overexpression and seeding are somewhat non-physiologic confounds induced by these conditions, and should be considered in interpretation of results. Most studies using BSCs culture them in 25% horse serum. The high concentrations of serum could reduce effectiveness of small molecules that bind tightly to albumin and lot to lot variation in the serum could introduce unknown confounds into various assays. Of course, future studies may allow the development of a more defined medium in order to refine the culture process. Finally, of importance when developing agents to act on the brain, BSCs do maintain vascular cells in culture [32] but cannot model an intact blood-brain-barrier so the absence of this must be considered when taking drug candidates through to further pre-clinical testing.

## BSC models to study amyloid-β pathology and amyloid-β-targeting therapies in AD

Both acute and organotypic BSC models have been established which have served to provide understanding of mechanisms underlying AD in a system which bridges single cell in vitro to whole animal in vivo studies. Acute slices have been used across AD research particularly for electrophysiology but will not be a focus of this review (see Fitzjohn et al., 2008 for a summary [4]). Herein, we seek to highlight previous BSC studies relevant to AD and indicate the further potential for BSCs to be used to explore some of the mechanisms underlying AD and to develop novel therapeutics.

Aβ deposition in extracellular plaques is a prominent molecular phenotype of AD [40]. Many current and past AD therapeutics aim to target Aβ pathology [41]. Earlier models to study Aβ relied on the addition of exogenous Aβ to induce AD-relevant changes to cultures of the hippocampus or cortex [42] with more recent BSC models prepared from transgenic mice expressing familial mutations in amyloid precursor protein or the presenilins [13, 38]. The first reported application of Aβ to organotypic hippocampal BSCs was in 1992, however this did not induce extracellular plaque deposition nor Aβ immunoreactivity [42]. More recent attempts to develop ex vivo models of Aβ deposition in BSCs from transgenic mice have resulted in a variety of outcomes, with most studies failing to demonstrate robust deposits. Cultures prepared from TgCRND8 mice did not result in amyloid pathology [38] while slice cultures prepared from 3xTg-AD mice show increased production of Aβ1–42 after 4 weeks in culture compared to 6 months in vivo demonstrating an accelerated progression of Aβ molecular pathology in this system in the absence of overt Aβ deposition [13]. Manipulation of 5xFAD BSCs, by microglial depletion drives some accumulation of Aβ; and replenishment of young but not aged 5xFAD microglia prevents this deposition [43]. In addition, BSCs biolistically transfected with human wild-type and mutated *APP* have been used to explore Aβ production, as well as, effects of γ-secretase inhibition [44]. Overall, these BSC models have shown subtle Aβ immunoreactivity (small diffuse deposits, limited/no Thioflavin S positivity, absence of dystrophic neurites, limited/no gliosis) but not plaque pathology recapitulative of what is observed in human disease. However, convincing plaque pathology and dystrophic neurites were reported in a seeded BSC model in 2016. BSCs seeded with aged APP23 or APPPS1 brain and supplemented with supraphysiological levels of Aβ1–40 develop Aβ plaques after 10 weeks in culture. Both of these variables were required in order to induce Aβ deposition that was Thioflavin S positive and colocalized with dystrophic neurites [45]. It is likely that this model, if reproduced, will enable a mechanistic insight into plaque development, Aβ seeding and the role of dystrophic neurites in AD. Alternatively, BSCs from adult APPSwDI mice show Thioflavin S-positive plaques alongside gliosis highlighting the preservation of in vivo pathology in ex vivo cultures from adult mice and have shown some utility to study anti-Aβ therapies albeit with limited neuronal survival [20]. In summary, several attempts from different groups at modeling Aβ molecular pathologies in BSCs show some useful applications but also future work will be required to reproduce these intriguing results and apply them to understanding their relevance in AD.

Synapse loss is a known correlate of dementia in AD [46, 47], yet this feature has been difficult to model and is not consistent amongst transgenic AD models. There is a limited understanding of why some models show this synaptic loss and others do not [48]. BSCs prepared from TgCRND8 mice show loss of synapses lending their potential to study synaptotoxicity or synaptoprotective compounds [38] but do not show any extracellular Aβ deposition even after culturing for 4 months which is longer than when plaques develop in vivo. Taken together, there are several useful BSC models which can be used to increase our understanding of the role of Aβ in disease and to develop appropriate therapies. These are summarized in Table 1.

**Table 1:** BSC models of Aβ physiology and pathophysiology

Human Pathology	Slice Culture Pathology	Reference(s)
Increased levels of Aβ production	BSCs from 3xTg-AD mice produce increased amounts of Aβ42 after 4 weeks in culture. BSCs from CRND8 mice also show elevated levels of Aβ42 from 2 weeks in culture. APP overexpression in non-transgenic rat cultures shows increased Aβ production by 3 days in culture.	[13, 38, 44]
Thioflavin S positive plaques	BSCs treated with APP23 or APPPS1 brain and supplemented with Aβ1–40 show Thioflavin S positive plaques. BSCs from adult APPSwDI show preserved Thioflavin S plaques in culture.	[20, 45]
Dystrophic neurites	BSCs treated with APP23 or APPPS1 brain and supplemented with Aβ1–40 develop dystrophic neurites.	[45]
Gliosis	BSCs from adult APPSwDI show preserved gliosis in culture.	[20]
Synapse loss	CRND8 BSCs develop synapse loss by 6 weeks in culture.	[38]

## Modelling tau molecular pathology in BSCs

Tau pathology is associated with dementia and neuronal loss in AD [49]. The development of in vitro models that recapitulate the molecular changes such as tau hyperphosphorylation and inclusion formation are also relevant to other neurodegenerative tauopathies [50–52].

There have been several reports of BSC models, which could be used to understand molecular pathways underpinning tau pathology or to assess tau-directed therapies. BSCs produced from postnatal tau transgenic mice expressing familial mutations in *MAPT* develop accelerated changes in tau similar to those found in AD and other tauopathies. BSCs prepared from JNPL3 mice and Htau mice develop conformationally altered and phosphorylated tau after 2 weeks in vitro [12]. These models highlight the utility for investigating AD-like changes in tau in BSC models particularly due to the acceleration of this phenotype compared to in vivo. JNPL3 mice typically only develop small amounts of phosphorylated and conformationally altered tau at 4 to 5 months of age [53] and at around 6 months of age in Htau mice [54]. This acceleration of disease-related changes in culture highlights the potential for faster translation, particularly for studying tau, as in vivo models are both time consuming and costly. Indeed, BSCs prepared from 3xTg-AD mice [55, 56] rapidly develop phosphorylated tau over 4 weeks in culture, whereas in vivo*,* phosphorylated tau is generally only reported at 12 to 15 months of age in this model [13]. This BSC model was used to identify a redistribution of tau to the membrane which was associated with an increased release of tau under pathological conditions. This may contribute to the propagation of tau pathology now recognized as a contributing factor to AD [57]. Stimulated neuronal tau release also occurred in non-transgenic BSCs highlighting the utility of BSC models to study mechanisms of physiological tau release [13]. Furthermore, BSCs prepared from mice expressing pro-aggregant Tau_RD_ΔK [58] related to frontotemporal dementia also accumulate phosphorylated and Thioflavin-S positive tau lending their use to screen for anti-aggregation therapeutics [59]. Additionally, synergistic and differential effects of Aβ and tau on dendritic simplification and synapse loss have been identified in BSCs providing mechanistic insight into this component of AD [60, 61]. We have also recently shown that by using rAAVs to express mutant *MAPT* in BSCs we can robustly and reproducibly develop an abundance of tau inclusions which in long-term culture leads to cell loss. Using this strategy we can easily compare different MAPT constructs in parallel and use this platform to screen for small molecule tau-modifying therapies [29]. Alternatively, the potential of tau transgenic BSC models to screen for tau antibodies has also been demonstrated [62, 63]. This is of particular interest as several anti-tau antibodies have already entered clinical trials, but much work is needed to determine the appropriate species of tau to target [64]. Overall, BSC models developed to date recapitulate key aspects of tau pathology and are summarized in Table 2. The majority of these models rely on overexpression of human tau proteins, similar to in vivo tau transgenic rodent models [39], and facilitate the study of pre-pathologies and pathologies relevant to tauopathies. Until a greater knowledge of the development of tau pathology and whether wild-type and mutant human tau with and without overexpression overlap, these models are likely valuable tools to expedite this understanding.

**Table 2:** BSC studies of tau physiology and pathophysiology

Human Pathology	Slice Culture Pathology	Reference(s)
Phosphorylated / conformationally altered tau	BSCs from 3xTg-AD, JNPL3, Tau_RD_ΔK and Htau mice all develop increased levels of phosphorylated tau from 1 to 4 weeks in culture. rAAV WT, S320F, P301L/S320F and A152T/P301L/S320F human tau transduced BSCs all accumulate phosphorylated tau by 28 days in vitro (DIV).	[12, 13, 29, 59]
Thioflavin S positive tau inclusions	rAAV P301L/S320F and A152T/P301L/S320F human tau transduced BSCs progressively develop Thioflavin S positive tau inclusions beyond 7 DIV. Tau_RD_ΔK BSCs accumulate Thioflavin S puncta from 25 DIV.	[29, 59]
Tau-induced cell loss	rAAV A152T/P301L/S320F human tau transduced BSCs develop cell loss by 2 months in culture.	[29]
Tau redistribution	BSCs from 3xTg-AD mice show an accumulation of tau at the membrane by 28 DIV. rAAV P301L/S320F and A152T/P301L/S320F human tau transduced BSCs show somatodendritic accumulation of tau by 28 DIV. Tau_RD_ΔK BSCs accumulate tau in the somatodendritic compartment.	[13, 29, 59]

## Exploring other mechanisms underlying AD in BSCs

As discussed above, BSC models can provide a system in which to study the molecular pathologies of Aβ and tau, as well as, synaptotoxicity. Furthermore, these BSCs can be used to explore other aspects of the disease and the role they play in the development or progression of pathology and to identify potential therapeutic targets.

Indeed, application of Aβ peptides to BSCs enables the investigation of the role of protein kinases implicated in AD [65]. Chiefly, the induction of GSK-3β [66, 67], the activation of ERK1/2 signaling [68] and the inactivation of AKT have [67] been observed in BSCs in response to incubation with the amyloid peptide Aβ25–35 or Aβ42 oligomers. The role of caspase activation in the AD brain is unclear; previous studies have suggested that caspases may promote the pathology of Aβ and the formation of NFTs [69]. Consistent with in vivo models, BSC models have demonstrated that amyloid peptide Aβ25–35 induces caspase-3 activation and apoptotic cell death [61, 68]. Understanding cell death and mechanisms of oxidative stress in response to Aβ and its prevention can also be explored in BSCs [70].

Inflammation is also an important and controversial feature of AD [71, 72] as its precise role in disease progression is complex and still unknown [73]. Since BSCs contain all the cell types of the CNS they are suitable models to investigate inflammatory mechanisms in AD. BSCs have been used to investigate the role of Apolipoprotein E on Aβ deposition [74, 75]. The role of microglia in plaque clearance has also been highlighted in BSC models. C1q protein and mRNA levels increased within BSCs in response to treatment with Aβ42 [76] indicating initiation of phagocytic activity of microglia. Pharmacological depletion of microglia in BSCs enables ‘plaque-like’ deposition after application of Aβ42, and subsequent replenishment of microglia decreases this ‘plaque-like’ reactivity [43]. Effects of the AD risk gene TREM2 have also been investigated in BSC models [77]. The benefits of several anti-inflammatory compounds against the degeneration of serotonergic, cholinergic, and dopaminergic neurons in Aβ42 treated BSCs have also been identified [78]. In addition, the changes astrocytes undergo in response to Aβ has also been highlighted in BSC models [79].

A novel model to study the degeneration of cholinergic neurons in the basal nucleus of Meynert has also been established [1, 80]. Cholinergic neurons are vulnerable in AD [1], and the authors established the possible benefits of trophic factors in this model to enable survival of cholinergic neurons.

## Using BSCs to determine the pathogenesis of other neurodegenerative proteinopathies

BSCs have been successfully applied to study several other neurodegenerative conditions and for therapeutic development. Neurodegenerative proteinopathies are commonly linked by the accumulation of misfolded proteins and associated downstream processes likely triggering neurodegeneration and potentially further propagation of aggregates. Aggregated α-synuclein found in Lewy bodies is a prominent feature of α-synucleinopathies including Parkinson’s disease (PD), Lewy body dementia and multiple system atrophy [81]. Delivery of mutant human synuclein using rAAV has been demonstrated to induce inclusions similar to those found in human disease in BSCs of the nigro-striatal circuit [82]. We have also shown induction of bona fide α-synuclein inclusion pathology with rAAV delivery of wild-type and mutant A53T human α-synuclein to BSCs of the hippocampus and cortex [29]. Mechanisms underlying the spread of α-synuclein pathology have also been explored in co-cultures of astrocytes and BSCs seeded with exogenous α-synuclein fibrils [83] highlighting the potential of this system to understand this aspect of α-synucleinopathies. Dopaminergic neuron loss, which is also a primary feature of PD can be induced chemically or progressively over time in rodent BSCs [11, 84].

Several genetic mutations have been identified which can lead to the development of amyotrophic lateral sclerosis (ALS). Mutations in these genes lead to accumulation of proteoplasmic inclusions such as superoxide dismutase-1 (SOD-1) inclusion pathology in the brain and spinal cord [85]. Spinal cord slice cultures prepared from transgenic G93A SOD-1 mice develop SOD-1 inclusions [86] and can also be induced with human SOD-1 seeds from spinal tissues [85]. TAR DNA binding protein 43 (TDP-43) inclusions and mutations are associated with the development of ALS and some frontotemporal dementias and ALS. BSCs prepared from human mutant TDP-43 expressing rats develop astrogliosis and microgliosis and have enabled the study of secreted factors which induce selective neuronal death [87]. TDP-43 inclusions have also been reported to be chemically induced in BSCs [88].

Huntington’s disease (HD) is characterized by the accumulation of huntingtin (HTT) aggregates and genetic mutations in HTT can drive disease [89]. Biolistic transfection of human wild-type and mutant huntingtin (HTT) causes accumulation of HTT aggregates in striatal and cortical BSCs and subsequent neurodegeneration. This recapitulates pathology reminiscent of human HD and can be used to screen for neuroprotective compounds [89]*.* Taken together, these examples summarized in Table 3 provide compelling evidence where BSC models have been successfully established to investigate proteinopathies and highlights the future potential of research using BSCs to understand mechanisms and develop therapeutics in these diseases.

**Table 3:** BSC studies models of proteopathic inclusion pathologies beyond Aβ and tau

Human Pathology	Slice Culture Pathology	Reference(s)
α-synuclein inclusions	rAAV expression of WT or A53T human α-synuclein in BSCs induces pser129 α-synuclein inclusions. rAAV expression of A53T human α-synuclein in nigrostriatal circuit BSCs also develop α-synuclein inclusions.	[29, 82]
SOD-1 inclusions	SOD-1 accumulation in spinal cord slice cultures from transgenic G93A SOD-1 mice from 3 weeks in culture. SOD-1 inclusions also inducible in G85R SOD-1 transgenic spinal cord cultures with human SOD-1 seeds from spinal tissues progressively over a 20 day incubation.	[85, 86]
TDP-43 inclusions	BSCs from rats expressing human mutant TDP-43 develop TDP-43 inclusions and associated micro- and astrogliosis from 10 DIV.	[87]
Huntingtin inclusions	Biolistic transfection of human wild-type and mutant huntingtin (HTT) in striatal and cortical rat BSCs results in the development of HTT inclusions by 7 DIV.	[89]

## What lies ahead for organotypic slice models of neurodegenerative proteinopathies

With the recent publication of several BSC models relevant to understanding the spectrum of molecular changes in AD and other neurodegenerative proteinopathies, it appears that these models can accelerate research in this area. How BSC models can potentially advance the field are discussed here.

## Future studies in AD

The propagation of tau has emerged as a potential contributor to the pathology observed in AD. On the basis of these findings, tau immunotherapies have rapidly entered clinical trials as it is posited that preventing this propagation could alleviate disease pathogenesis. It is still unclear as to which is the main species of tau which propagates in AD and the mechanisms behind this propagation [57, 64] and these are questions which could be explored in BSC models. The relationship between tau and neurodegeneration is likely to be critical in our understanding and treatment of tauopathies like AD. Overexpression of human tau bearing three *MAPT* mutations leads to rapid inclusion formation and subsequent cell death in slices [29]. It is plausible that BSCs can be used to gain insights into these enigmatic aspects of the disease and facilitate understanding of the relationship between tau and cellular dysfunction in tauopathies. As described earlier, BSCs can be used to identify differences between the release of pathological and physiological tau [13]. In addition, the function of physiological extracellular tau and its spread also remains unclear. BSC models lend themselves to study both of these questions in a more intact system than cell models of propagation but in an easier, more rapid manner than exploring these questions in vivo*.* Similarly, it has recently emerged that limited *MAPT* mutations are permissive to seeding in a cell-based assay [90], it would be interesting to identify whether this is also the case in a more intact system and whether a seeded BSC model of tauopathy can be developed. The concept of using compounds or antibodies to remove or clear this pathological extracellular tau could be explored in BSCs [62, 63]. As it is unknown how similar the pathways to aggregation are between wild-type tau and that containing familial mutations, BSCs may provide an optimal platform for attempting aggregation of wild-type tau using seeding or through manipulation of kinases and pathways involved in aggregate clearance. Until it is fully understood whether mutant and wild-type tau follow a common aggregation pathway, it will be unclear whether attempting to target mutant tau will translate to blocking wild-type tau aggregation, as in sporadic AD. What is more clear is that mutant tau overexpression leads to the formation of tau inclusions that are indiscernible from those found in sporadic AD brain in terms of phosphorylation status and structure as seen by electron microscopy [29]. Therefore, it is likely that the downstream pathways and cellular dysfunction will be well modeled using this system. Additionally, the role of Aβ aggregation in the spread and development of tau pathology is less clear and could also be investigated in BSC models.

The precise role of innate immune activation and neuroglia in AD [71, 72] still needs to be determined. Some established BSC models to study the role of inflammation were highlighted earlier. It is unclear as to how inflammation and neuroglia should be tuned in AD, and as BSCs contain all of these cell populations they could be exploited to explore this aspect of the disease. Similarly, the majority of proteinopathies likely occur due to complex interactions between the multiple cell types in the brain. BSCs containing all these relevant cell types should enable the modeling of interplay between them and their role in disease.

## Combining rAAV tools and other technologies with BSCs

Transgenic animals have been invaluable to study molecular mechanisms underpinning proteinopathies. BSCs produced from neonatal transgenic mice overexpressing AD-related genes preclude the aging of these mice whilst developing disease-relevant pathologies [12, 13, 45]. However, the production of these transgenic mice is expensive, requiring significant time and resources to age these mice to observe pathologies relevant to AD and other proteinopathies. rAAVs provide an efficient gene delivery platform and important tool for neuroscience research. We have shown that rAAV-mediated gene delivery enables the transduction of genes of interest to non-transgenic BSCs to induce abundant AD or PD inclusion pathology [29]. Mutations in human *APP,* the presenilins or *MAPT* known to cause familial AD and tauopathies, respectively, or a combination of these can easily be delivered to BSCs from transgenic or non-transgenic mice in order for us to understand mechanisms related to AD and develop novel BSC models. In addition, several genes which increase susceptibility to sporadic AD have been identified in recent years [91], and their role in the progression of AD remains unclear. These risk genes could easily be expressed using rAAVs in BSCs to assess any contribution to the development of the disease. This approach can also be employed to study other proteopathic inclusions associated with other neurodegenerative diseases and in novel therapeutic development for these conditions. Similarly, advances in the development of CRISPR-Cas9 and zinc finger nuclease gene editing [92] and RNAi gene silencing [93] rAAV vectors could be applied to BSCs and will likely be useful tools in determining genetic contributions to proteinopathies.

Furthermore, the majority of proteinopathies likely occur due to complex interactions and relationships between all the CNS cell types. We have shown that using rAAVs in BSCs containing all these cell types we can selectively manipulate neurons, astrocytes, microglia, oligodendrocytes or combinations of these allowing us to explore cell autonomous and non-cell autonomous roles in proteinopathies [29].

Similarly, further rAAV technology has been developed to enable expression of functional reporter genes such as voltage sensors, calcium sensors, retrograde tracers, expression of channelrhodopsins to facilitate optogenetic experiments, or designer receptors exclusively activated by designer drugs (DREADDs) as chemogenetic tools [94–99]. In combination with BSCs and improved imaging techniques it is likely these rAAV tools could rapidly inform our understanding of functional neuronal circuitry and any changes associated with proteinopathies. A summary table of some examples of rAAV tools already used in BSCs and for potential use in BSCs is provided in Table 4. Promoters and capsids are typically interchangeable in order to obtain target expression so the promoters and capsids provided are likely not the only examples or options [29].

**Table 4:** rAAV tools which can be applied to studies of neurodegenerative proteinopathies in BSCs

rAAV Tool	Promoter(s)	Capsid(s)	Experimental model	Reference(s)
Non-Specific global gene expression	hybrid cytomegalovirus enhancer/chicken β-actin (hCBA)	2/1, 2/2, 2/6, 2/8, 2/9	BSCs	[29]
Microglial expression	CD68	2/6 with three mutations Y731F/Y705F/T492 V (TM6)	BSCs	[29]
Neuronal expression	CamKII or MAP-2	2/6, 2/8	BSCs	[29]
Oligodendrocyte expression	MBP	2/8	BSCs	[29]
Astrocyte expression	GFAP	2/8	BSCs	[29]
Calcium-Sensing e.g. GCaMP6 or REX-GECO1	Synapsin, chicken β-actin (CAG) or cytomegalovirus (CMV)	2/1	In vivo rodent, BSCs	[95, 96]
Voltage-Sensing e.g. Archon1 or Voltron	CamKII or CAG	–	Acute BSCs, Primary Hippocampal Neurons	[94]
Retrograde Tracing	CMV or Synapsin	2-retro (Mutant r5H6 (insert LADQDYTKTA + V708I + N382D))	In vivo rodent	[98]
Optogenetics e.g. Channelrhodopsin-2	CAG	2/1	In vivo rodent, Acute BSCs	[99]
Chemogenetics e.g. DREADDs	Synapsin	2/8	In vivo rodent, Acute BSCs	[97]
Recombination e.g. Cre	CMV	2/2	In vivo rodent	[100]
Gene Editing e.g. CRISPR/Cas9	CMV	DJ, 2/9	In vivo rodent	[92]
Gene Silencing e.g. shRNA	Modified CMV, H1	2/1	In vivo rodent	[93]

BSCs represent an accessible system for methodologies which may typically be more difficult or invasive to execute such as long-term live imaging. Imaging, in combination with rAAV tools, should be exploited in future studies to increase our understanding of proteinopathies and changes over time. It is likely this approach would rapidly yield significant information about the roles of cellular dysfunction in proteinopathies, as well as, increasing our understanding of the role of protein aggregates in this. It will also be important to carefully compare these findings to the more difficult imaging studies in in vivo animal models to confirm their utility.

## BSC models from adult mice

As proteinopathies typically develop only in adults with mature, long-existing neurons [101] it would be ideal to examine disease pathogenesis in BSCs containing aged neurons. There have been several efforts from different groups to prepare BSCs from adult mice but these have all shown limited success in preserving large numbers of neurons. Vast cell death (less than 20% cell survival) [21, 102] has been reported as well as a low yield, with only 5–10% of cultures surviving to a month in culture [14, 102, 103]. Some groups have reported survival of some cells in long-term cultures of adult brain [19] but still with limited viability of neurons [20]. This is even with significant methodological alterations such as maintaining in lower temperatures, different culture mediums, carbogen atmospheres and decreased thickness [19–21]. It has been difficult to ascertain why adult BSCs do not survive culture as well as BSCs from neonates, there has been some speculation that neonatal cultures are plastic enough to remodel after axons are severed during preparation but adult BSCs cannot recover from this axonal degeneration [18]. To test this, we prepared BSCs from Sarm1^−/−^ mice which were reported to show resistance to axon degeneration [104], however adult BSCs from these mice showed no increased survival compared to controls suggesting differential Sarm1 and non-Sarm1 mediated pathways of degeneration. Poor adult BSC survival may also be related to their post-mitotic phenotype or greater vulnerability to the culture environment [102].

Taken together, there are still many challenges to overcome in order to maintain adult BSCs with a large proportion of viable neurons. However, should these barriers be overcome and a consistently viable BSC preparation from adult proteinopathy mice were possible, it would enable us to screen compounds for proteinopathies and target molecular pathways underlying these diseases in these aged neurons with aged proteopathic inclusions.

## Therapeutic screens in BSCs

BSCs provide an intact, physiologically relevant system to study compounds to target the CNS. In the setup we use, 12 to 36 compounds can be screened in cortical BSCs prepared from one animal and subsequently analyzed biochemically or histologically. This lends to a relatively low throughput system but is a system more representative of in vivo conditions compared to the cell lines usually used in high-throughput screening for drug candidates. Furthermore, compounds with an unknown mode of action can be studied in this integrated model rather than screening compounds for a particular mode of action typical of high-throughput screens [105]. Indeed, this method of screening has already been used to identify candidate leads for neuroprotection [105]. Future work should also be focused on developing a more defined culture medium as the majority of cultures reported use 25% horse serum which may not be amenable to the study of some compounds. The concept of using compounds or antibodies to remove or clear pathological aggregates can also be explored in BSCs [62, 63].

## Conclusions

BSC models provide a powerful bridge combining the accessibility and control of in vitro approaches whilst preserving the physiology, cytoarchitecture and synaptic integrity of an intact brain. It is clear that research involving BSCs can offer a beneficial insight into the molecular mechanisms underlying the development and progression of neurodegenerative proteinopathies. In addition, BSCs can be exploited to develop and pre-screen novel therapies to prevent or treat these diseases, likely enabling a faster translation to the clinic. With advances in other technologies such as viral vector delivery and imaging methods that can complement BSCs; this is an exciting platform to be used in proteinopathy research.

## Data Availability

Not applicable.

## Abbreviations

AD: Alzheimer’s disease
ALS: Amyotrophic lateral sclerosis
Aβ: Amyloid-β
BSC: Organotypic brain slice culture
CAG: Chicken β-actin promoter
CMV: Cytomegalovirus promoter
DIV: Days in vitro
DREADDs: Designer receptors exclusively activated by designer drugs
hCBA: Hybrid cytomegalovirus enhancer/chicken β-actin promoter
HD: Huntington’s disease
Htt: Huntingtin
LB: Lewy body
MAPT: Microtubule-associated protein tau
PD: Parkinson’s disease
rAAV: Recombinant adeno-associated virus
SOD-1: Superoxide dismutase-1
TDP-43: TAR DNA binding protein 43
TM6: rAAV2/6 with three mutations Y731F/Y705F/T492 V
